# Myocardial Metabolic Response Predicts Chemotherapy Curative Potential on Hodgkin Lymphoma: A Proof-of-Concept Study

**DOI:** 10.3390/biomedicines9080971

**Published:** 2021-08-06

**Authors:** Cecilia Marini, Matteo Bauckneht, Anna Borra, Rita Lai, Maria Isabella Donegani, Alberto Miceli, Cristina Campi, Vanessa Cossu, Daniela Schenone, Silvia Morbelli, Stephane Chauvie, Michele Piana, Andrea Gallamini, Gianmario Sambuceti

**Affiliations:** 1 Nuclear Medicine, IRCCS Ospedale Policlinico San Martino, 16132 Genoa, Italy; cecilia.marini@unige.it (C.M.); silviadaniela.morbelli@hsanmartino.it (S.M.); piana@dima.unige.it (M.P.); sambuceti@unige.it (G.S.); 2 CNR Institute of Molecular Bioimaging and Physiology (IBFM), 20090 Milano, Italy; 3 Department of Health Sciences (DISSAL), University of Genoa, 16132 Genoa, Italy; annaborra@hotmail.it (A.B.); isabella.donegani@gmail.com (M.I.D.); albertomiceli23@gmail.com (A.M.); vane.6291@gmail.com (V.C.); 4 Department of Informatics, Bioengineering, Robotics and Systems Engineering (DIBRIS), University of Genoa, 16128 Genoa, Italy; lai@dima.unige.it; 5 LISCOMP Lab., Department of Mathematics (DIMA), University of Genoa, 16132 Genoa, Italy; campi@dima.unige.it (C.C.); daniela.schenone25@gmail.com (D.S.); 6 Department of Medical Physics, S. Croce Hospital, 12100 Cuneo, Italy; chauvie.s@ospedale.cuneo.it; 7 Research and Clinical Innovation Department, Antoine Lacassagne Cancer Center, 06189 Nice, France; andrea.gallamini@nice.unicancer.fr

**Keywords:** Hodgkin lymphoma, chemotherapy, fluorodeoyglucose, positron emission tomography

## Abstract

Genome sharing between cancer and normal tissues might imply a similar susceptibility to chemotherapy toxicity. The present study aimed to investigate whether curative potential of doxorubicin, bleomycin, vinblastine, and dacarbazine (ABVD) is predicted by the metabolic response of normal tissues in patients with Hodgkin lymphoma (HL). METHODS: According to current guidelines, 86 patients with advanced-stage (IIB-IVB) HL, prospectively enrolled in the HD0607 trial (NCT00795613), underwent 18 F-fluorodeoyglucose PET/CT imaging at diagnosis and, at interim, after two ABVD courses, to decide regimen maintenance or its escalation. In both scans, myocardial FDG uptake was binarized according to its median value. Death and disease relapse were recorded to estimate progression-free survival (PFS) during a follow-up with median duration of 43.8 months (range 6.97–60). RESULTS: Four patients (4.6%) died, while six experienced disease relapse (7%). Complete switch-off of cancer lesions and cardiac lighting predicted a favorable outcome at Kaplan–Mayer analyses. The independent nature and additive predictive value of their risk prediction were confirmed by the multivariate Cox regression analysis. CONCLUSION: Susceptibility of HL lesions to chemotherapy is at least partially determined by factors featuring the host who developed it.

## 1. Introduction

According to current guidelines [1], treatment of Hodgkin lymphoma (HL) implies the administration of two chemotherapy cycles with doxorubicin, bleomycin, vinblastine, and dacarbazine (ABVD), followed by a PET/CT study with 18 F-fluorodeoyglucose (FDG) (interim PET) [2]. This evaluation represents the most powerful ABVD outcome predictor, even superseding other well-known predictive factors [3,4], and thus aims to identify the need for treatment escalation to the more aggressive regimen using bleomycin, etoposide, doxorubicin, cyclophosphamide, vincristine, procarbazine, and prednisone (BEACOPP) that provides a higher disease-free survival, but at the cost of substantially higher toxicity in terms of death and secondary malignancy [5].

In the clinical practice, PET/CT criteria utilized for therapeutic decision are obviously focused on the response of number, size, and metabolic activity of lymphoma lesions. However, this standardized procedure intrinsically allows the evaluation of chemotherapy effect on normal tissues, and particularly on the heart [6]. The relevance of this assessment has been confirmed by the evidence that cardiac FDG uptake increases at interim PET in more than one half of HL patients and predicts the subsequent risk of developing cardiotoxicity [7,8]. This heterogeneous response might reflect a number of contaminant factors. Yet, it might also indicate the susceptibility of any given patient to ABVD toxicity. Both normal tissues and neoplastic cells share a large part of the genic asset and are exposed to the same factors able to modulate their sensitivity to chemotherapy toxicity. Accordingly, the curative potential of ABVD might be at least partially envisaged by the treatment effect on the metabolic pattern of normal tissues despite their absent involvement in cancer progression.

Testing this hypothesis in a selected population submitted to standardized imaging protocols and followed for an adequate timespan, here we show that cardiac metabolic pattern at interim PET actually predicts the risk for HL relapse or complication.

## 2. Materials and Methods

### 2.1. Study Design

The selection of studied patients exploited the database of the HD0607 trial [2]. This study included 782 patients with histopathologic diagnosis of classic HL, aging 18 to 60 years, Ann Arbor stage IIB to IVB, measurable international prognostic score (IPS), and able to sign the written informed consent form. The exclusion criteria were: concomitant or previously treated (<5 years) neoplasia, psychiatric disorder, impaired cardiac (ejection fraction < 50%) and renal (creatinine clearance < 60 mL/min) functions, HIV or any other active uncontrolled infection, pregnancy, and uncompensated diabetes.

According to the study design, all patients were submitted to staging PET/CT (baseline PET) before chemotherapy. Treatment started with two ABVD cycles (administered at standard doses) and was followed by an early PET/CT evaluation (interim PET) acquired using the same scanner of the baseline evaluation. Interim PET was analyzed according to the Deauville scoring system (DSS) that indicated the maintenance of ABVD regimen (for scores 1–3) or its escalation to BEACOPP scheme (for scores 4–5).

Complete remission was defined according to current guidelines [2,9,10]. Similarly, follow-up was prolonged for five years and implied ambulatory visits once every six months for the first 36 months, and each year thereafter [2,9,10].

### 2.2. Image Analysis

To estimate total metabolic tumor volume (MTV), the maximum standardized uptake value (SUVmax) of every active HL lesion was obtained in the transaxial view of attenuation-corrected FDG images. A volume of interest was then drawn using an SUV-based automated contouring program with the isocontour thresholding set at >40% of the SUVmax. MTV was thus defined as the sum of all lesion volumes.

At interim PET/CT, DSS was estimated according to standard criteria [11]; score 1 was defined locally, while scores ≥ 2 called for a central revision by three expert reviewers.

Myocardial FDG uptake was assessed as previously described [7,8,12,13] in both baseline and interim PET/CT. Briefly, a volume of interest was identified on the visible left ventricular myocardium on PET images, while CT series were used as a reference only in case of absent cardiac uptake. Average myocardial SUV was estimated in both staging and interim PET scans.

Muscular FDG uptake was analyzed in both psoas muscles, the large part of whose volume is systematically included in the standard PET/CT acquisition, while their contractile activity is minimized in the resting supine position maintained by the patient during the radiotracer biodistribution phase and PET/CT images acquisition. To this purpose, we applied a computational strategy that represents a slight modification of the one adopted for the assessment of the bone marrow metabolic activity [14,15]. CT images were visualized to determine the psoas insertion starting at the soma of D12. Then, a software tool developed in our lab utilized histogram equalization, edge detection, and an α-shape algorithm to segment the psoas volumes whose caudal limit was set as the plane crossing the L5–S1 junction. Eventually, the recognized region in each slice allowed the construction of a binary mask (1 inside the psoas domain; zero elsewhere), which was multiplied against the PET data to compute average FDG SUV in the defined psoas volume.

### 2.3. Statistical Analysis

Continuous variables are described as mean  ±  standard deviation (SD) and categorical data as percentages. Comparison between groups or within the same group at different times were performed using the Student’s t-test for unpaired and paired data, respectively. Correlation between different variables was tested by the least squares method.

Survival analyses were focused on the incidence of death or composite endpoint, including death and disease relapse, to estimate overall survival (OS) and progression-free survival (PFS), respectively. Follow-up time started at the date of interim PET and, for patients experiencing disease relapse, it was censored at that date.

First, for descriptive purposes, time-to-event distributions were estimated for all 86 patients using the Kaplan–Meier method and compared with the log-rank test. To this purpose, the continuous variables MTV, myocardial SUV, and psoas muscle SUV were binarized according to the 50th percentile. Since OS was only predicted by DSS at interim PET/CT, multivariate analysis was only performed considering PFS. To this purpose, all variables were tentatively included in a multivariate Cox’s model by means of a step-back (backward) procedure, based on the likelihood ratio test; a *p*-value < 0.15 was required for the inclusion of a variable in the model.

A *p*-value < 0.05 was considered statistically significant. The used statistical software was SPSS Inc., Advanced Models 15.0 (Chicago, IL, USA).

### 2.4. Study Approval

We reviewed the clinical research forms of the HD0607 trial (Eudract code 2007-007168-94, Clinicaltrial.gov (accessed on 1 July 2021) identifier NCT00795613) [2]. The protocol of the study was approved in 1 June 2008 by the Santa Croce e Carle Hospital institutional review board (identification number Emato 16). The study was conducted in accordance with the International Conference on Harmonization for Good Clinical Practice guidelines and the Declaration of Helsinki; it requested the preliminary signature of the written informed consent, and it was approved by the ethics committees of all the participating centers.

## 3. Results

### 3.1. Clinical Characteristics and Follow-Up Data

Among the 782 subjects included in the original database, we selected the datasets of the 86 patients with image format compatible with the software used for analysis. As shown in Table 1, the main clinical characteristics of the population included in the present sub-analysis were representative of the entire cohort. Images with DSS 1–3 were observed in 67/86 (78%) patients, who thus prosecuted ABVD regimen. By contrast, a 4–5 DSS escalated the subsequent regimen to BEACOPP in the remaining 19 (22%) patients.

### 3.2. FDG Uptake Response to ABVD in the Heart and Skeletal Muscle

**Figure 1:**
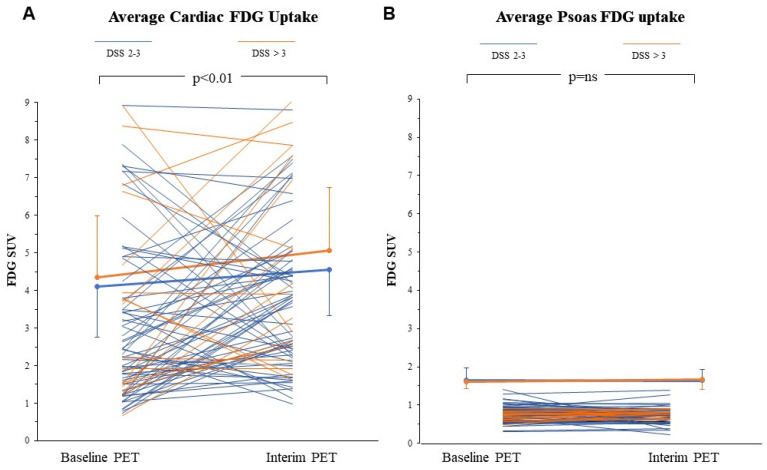
Individual and average cardiac (thick lines) SUV (**A**) increased to a comparable extent from baseline to interim PET in patients with acceptable response to ABVD regimen (DSS 2–3, blue lines), and in those in which therapy scheme was escalated to BEACOOP because of a DSS > 3 (orange line). This systematic response was not observed in psoas muscles (**B**) also because of the markedly lower SUV of this tissue. ns = not significant.

**Figure 2:**
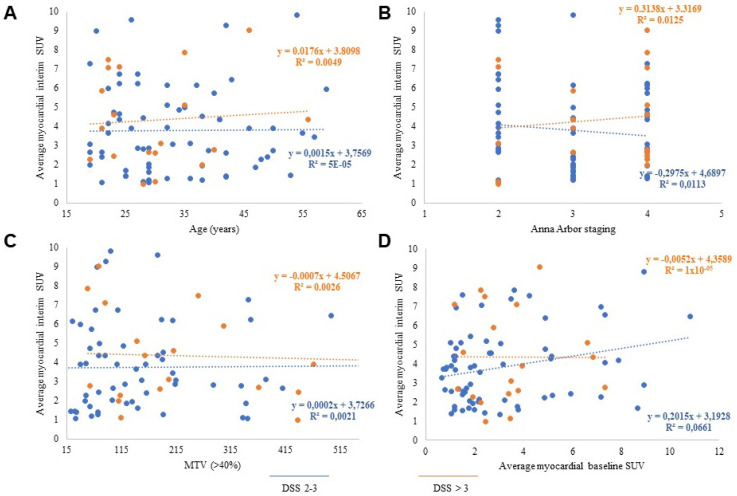
Average cardiac SUV at interim PET was independent of age (**A**), Ann Arbor staging at baseline (**B**), disease bulk evaluated by MTV (**C**), and the corresponding tracer retention before ABVD (**D**). The correlation was absent in both patients with DSS 2–3 (blue dots and lines) and DSS > 3 (orange dots and lines).

In the whole population, myocardial SUV increased from baseline to interim PET (from 3.2 ± 2.1 to 4 ± 2.0, respectively, *p* < 0.01) (Figure 1A). An increase, defined by a ratio SUV interim/SUV baseline > 1, was observed in 52/86 patients (60%). At chi-square analysis, this response was independent of age (*p* = ns, odds ratio 0.91; 95% CI: 0.38–2.15), and tumor bulk as evaluated by metabolic tumor volume (MTV) (*p* = ns, odds ratio 1.62; 95% CI: 0.68–3.86). Similarly, myocardial SUV at interim PET was independent of all these variables, and of the Ann Arbor staging (Figure 2A–C).

Similarly, it was completely unrelated to the myocardial metabolic pattern observed at baseline PET before chemotherapy administration (Figure 2D). Finally, it was also independent of chemotherapy curative potential, since myocardial SUV at interim PET was similar in patients with DSS 1–3 or 4–5 (3.9 ± 1.9 vs. 4.3 ± 1.5, respectively, *p* = ns) (Figure 1A).

Differently from the myocardium, the metabolic pattern of psoas muscles was not affected by chemotherapy. Indeed, average FDG SUV was virtually identical at staging and interim PET (0.76 ± 0.2 vs. 0.76 ± 0.2, respectively, *p* = ns) (Figure 1B). Again, no significant difference could be observed between patients with negative (DSS 1–3) or positive (DSS 4–5) interim PET.

Accordingly, these data indicate that the first two ABVD courses can have a direct effect on the metabolic pattern of normal host tissues not involved by the disease. However, this effect is not homogeneously distributed throughout the host and is best appreciated in the heart, suggesting the local occurrence of a series of events able to modify the myocardial avidity for FDG.

### 3.3. Predictive Power of Cardiac FDG Uptake

According to the selection criteria, ABVD treatment scheme was completed in all patients with DSS 1–3 at interim PET, while the BEACOOP scheme was completed in all patients scored as 4–5 at the same time point. Follow-up data were available in all patients, with a median duration of 43.8 months (range 6.97–60). During this interval, among the 86 studied patients, four patients (4.6%) died, while six experienced disease relapse (7%).

**Figure 3:**
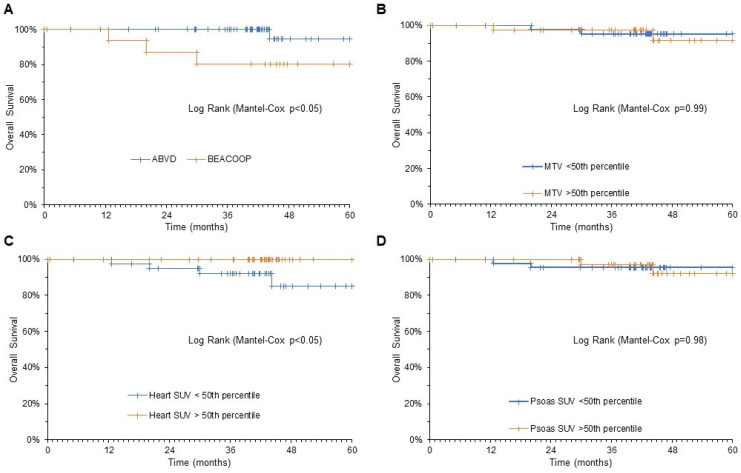
Kaplan–Meier analysis of OS. Mortality rate was predicted by the need of therapy escalation according to DSS (**A**) and cardiac SUV at interim PET (**C**). By contrast, disease bulk evaluated by MTV (**B**) and skeletal muscle SUV (**D**) were devoid of this predictive power.

As shown in Figure 3, OS was predicted by the residual disease at interim PET. Indeed, a DSS 1–3, and thus the possibility to complete the ABVD scheme, eventually resulted in a mortality rate of 1.5% throughout the follow-up. By contrast, a higher DSS, and thus the need to upgrade treatment to BEACOPP protocol, was associated with a mortality rate of 15.7% during the same interval (*p* < 0.05). A similar predictive power also applied to myocardial metabolic pattern at interim PET/CT. Indeed, OS at five years was 100% and 85% in patients with heart SUV > 50th percentile or below this threshold, respectively (*p* < 0.05).

Both Ann Arbor staging and MTV did not significantly predict OS (Figure 3). Likewise, overall mortality rate was not predicted by psoas SUV at the same time (Figure 3).

**Figure 4:**
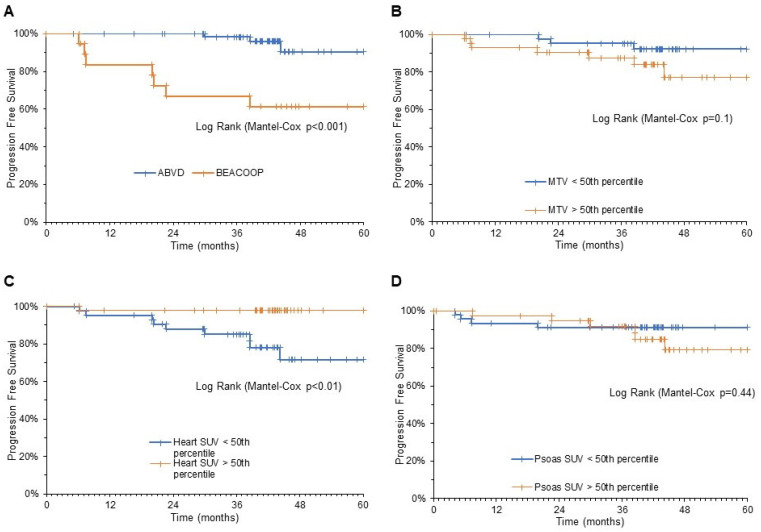
Kaplan–Meier analysis of PFS. Event-free survival was predicted by the need of therapy escalation according to DSS (**A**) and cardiac SUV at interim PET (**C**). By contrast, disease bulk evaluated by MTV (**B**) and skeletal muscle SUV (**D**) were devoid of this predictive power.

When the composite endpoint was considered (death and disease relapse), PFS was significantly predicted by DSS and by myocardial metabolic pattern at interim PET; patients with DSS 1–3 and patients with myocardial SUV > 50% percentile at interim PET showed a significantly longer PFS with respect to the remaining patients (Figure 4).

Univariate analysis defined DSS as the only significant predictor of OS. By contrast, predictive prognostic factors for PFS were Ann Arbor staging, DSS, and average myocardial SUV at interim PET. However, the multivariate analysis identified an additive and independent predictive power for PFS only for the myocardial SUV and for the DSS-triggered escalation to BEACOOP therapy (Table 2).

## 4. Discussion

The present data indicate that cardiac metabolic response to the first two ABVD courses predicted the anticancer effect of chemotherapy and stratified the five-year risk for the combined endpoint (death and disease relapse) in HL patients. This predictive power was additive and independent with respect to the response of cancer lesions. More importantly, risk was predicted by a divergent response of FDG uptake in these two districts: favorable outcome was anticipated by the tumor switch-off as opposed to myocardial lighting. Thus, these observations indicate that HL susceptibility to ABVD effect is at least partially dependent upon genetic or phenotypical features of the host who developed it.

### 4.1. Tumor Shutdown vs. Cardiac Lighting

Our comprehensive approach to PET/CT images was focused on the heart and the skeletal muscles because both tissues are barely involved in HL. By contrast, bone marrow was not evaluated because of either its possible involvement by disease or the contamination caused by the metabolic response to its hyperplastic reaction to cytoreductive chemotherapy effect [16,17,18].

Cancer response to the chemotherapy followed the expected behavior with FDG uptake decreasing, though to a different degree, from baseline to interim PET in virtually all lymphoma lesions. According to current guidelines, this response represents the expected curative effect of the two administered ABVD courses and their capability to induce the death of neoplastic cells, followed by a reduction in the local inflammatory response [19]. Therefore, the impaired tracer retention seems to represent the combination of two main mechanisms. On one side, it might derive from the decrease in number or density of metabolically active cancer cells caused by the cytotoxic action of chemotherapy. On the other side, it might also reflect a metabolic switch off caused by either the arrest of proliferating activity or the reduction in the inflammatory lesion environment [20,21].

ABVD effect on normal tissues was expectedly more variable: FDG uptake was left unchanged in skeletal muscles, while it was markedly increased in the heart. Both tissues were selected because of their unlikely involvement by HL elements both at baseline and, *a fortiori*, after the two ABVD cycles. Accordingly, the cardiac lighting should represent the result of a series of events selectively triggered by the chemotherapy on the myocardium, and independent of HL burden and location. This response occurred in the majority (60%) of patients, but not in the whole population, confirming the clinical experience and allowing the analysis about the impact of host metabolic pattern on HL evolution.

### 4.2. Mechanisms Underlying Cardiac Lighting

The metabolic activation of the heart, observed at interim PET, is not a novel finding and confirms previous observations about its capability to predict the cardiotoxic effect of the ABVD component doxorubicin [8,22]. Under many viewpoints, the drug mechanism of action might at least partially explain this finding. Indeed, anticancer potential of doxorubicin at least partially reflects its capability to bind topoisomerases as to impair the simultaneous cut of the two DNA strands, resulting in the death of highly proliferating cancer cells [23]. This same molecular target seems to characterize the myocardium due to the high cardiomyocyte expression of the mitochondrial topoisomerase isoform 1. This enzyme is dedicated to the control of mitochondrial DNA and is strikingly hampered by the preferential doxorubicin accumulation in this organelle [24,25]. The consequent impairment of oxidative phosphorylation inevitably enhances the generation of reactive oxygen species and is particularly relevant in the heart, due to the aerobic metabolism of the myocardium and the local combination of high mitochondrial density and relatively low levels of antioxidant-producing enzymes [26].

The link between redox stress and FDG uptake was obviously not investigated in the hearts of the present population. Nevertheless, Bauckneht and colleagues [22,27] previously documented that the retention of the radioactive glucose analogue is strictly and directly related with the degree of oxidative damage and the amounts of reactive oxygen species measured in the myocardium of mice treated with therapeutic doses of doxorubicin. In agreement with similar studies in cancer, brain, and skeletal muscles, this correlation was explained by the increased expression and activity of the FDG-processing enzyme hexose-6P-dehydrogenase that triggers a pentose phosphate pathway (PPP) selectively located within the endoplasmic reticulum. This observation thus nicely fits with the notion that PPP is the main pathway fueling the NADPH reductive power needed by glutathione-dependent antioxidant responses, as well as with the doxorubicin capability to induce a severe endoplasmic reticulum stress [22,28,29].

The selectivity of heart lighting thus fits both the high susceptibility of the myocardium to the doxorubicin-induced redox stress and the link between oxidative damage and FDG uptake. In agreement with this observation, the present evidence about the protective effect of cardiac lighting on PFS faces our previous observation of an increased risk for cardiotoxicity in patients with the same response [8]. Therefore, the direct proportionality between doxorubicin curative potential and (cardio)toxic risk seems to indicate that the sensitivity to doxorubicin action is shared by cancer and its host.

### 4.3. Limitations

The main limitation of the present study is represented by the small number of studied patients. This was caused by the need to select images stored with a format compatible with our software. However, this technical constraint provided us with a population whose features at the enrollment were remarkably similar with respect to the original database. Likewise, OS and PFS were comparable between our study and the previous large-scale one.

As a second consideration, the retrospective nature of our investigation did not permit the application of a standardized diet regiment to avoid the interference of nutritional condition on myocardial FDG uptake. However, the invariance of tracer retention in the skeletal muscles strongly indicates a negligible role of the interference of dietary regimen on measured cardiac SUV. Nevertheless, optimizing the clinical potential of this analysis would probably need to precede all PET/CT scans in HL patients by a lipid-rich dietary regimen in order to switch off myocardial glucose consumption in a standardized fashion [30]. A similar consideration applies to the potential interference by the intercurrent treatment with cardioactive therapies. However, criterion for study entry was the absence of cardiac disease in the clinical history, as well as the evidence of left ventricular dysfunction, thus potentially reducing the impact of this confounding factor.

Accordingly, these limitations do not permit the consideration of myocardial SUV at interim PET as a parameter able to modify therapeutic planning in the clinical setting. To this purpose, reproducing this observation in a larger database would be needed. Yet, the same limitations do not hamper the confirmation of our hypothesis, i.e., the relevance of pre-existing genic and phenotypic factors related to the host and able to determine cancer susceptibility to ABVD curative effect.

In conclusion, the link between increased FDG uptake and subsequent development of cardiotoxicity after anthracycline treatment had been already documented. Extending that observation, the present data indicate that the same signal also represents a marker of cancer susceptibility to the same treatment. This evidence thus confirms that the cytotoxic effect of ABVD regimen is shared by the cancer and the ecosystem—the host—in which the disease arose. If confirmed, this hypothesis might configure PET/CT imaging as a fundamental tool in personalizing the therapeutic strategy to HL and, probably, to other tumor candidates to chemotherapy.

## Figures and Tables

**Table 1:** Clinical characteristics of evaluated patients and comparison with the original source database.

		Present Study	HD0607 Trial
**Number**		86	782
**Median age, years (range)**		29.5 (19–59)	31 (14–60)
	≥50 years	8 (9%)	10%
**Sex**			
	Male	41 (48%)	49%
	Female	45 (52%)	51%
**WHO activity index**			
	0–1	77 (89%)	90%
	>1	9 (11%)	9%
**Ann Arbor Staging**			
	II	26 (30%)	26%
	III	30 (35%)	32%
	IV	30 (35%)	32%
**International Prognostic Score (IPS)**			
	0–1	31 (37%)	37%
	2–3	46 (53%)	51%
	>3	9 (10%)	12%
**Large Nodal mass**			
	<5	35 (41%)	42%
	5–7	14 (16%)	18%
	8–10	19 (22%)	20%
	>10	18 (21%)	20%
**B symptoms**		64 (75%)	81%

**Table 2:** Prediction of PFS.

Variable	Strata		Num of Patients	Num of Events	%	Univariate			Multivariate		
						Hazard Ratio	95% CI	*p*	Hazard Ratio	95% CI	*p*
	Total		86	10	12%						
**Age**											
	<median		43	4	9%	1 (ref)					
	>median		43	6	14%	1.32	0.37–4.70	0.67			
**Ann Arbor Staging**											
	2		28	3	11%	1 (ref)					
	3		28	1	4%	0.38	0.04–3.71	0.41			
	4		30	6	20%	1.99	0.49–7.96	0.33			
**IPS Score**											
	1		26	3	12%	1 (ref)					
	2		27	2	7%	2.74	0.53–14.22	0.23			
	3		24	4	17%	2.01	0.33–12.08	0.44			
	4		9	1	11%	-	-	0.98			
**MTV at baseline PET**											
	<median		43	3	7%	1 (ref)					
	>median		43	7	16%	2.64	0.69–10.24	0.15			
**Deauville Score at Interim**											
	1–3		67	3	4%	1 (ref)			1 (ref)		
	4–5		19	7	37%	8.9	2.29–34.83	**0.002**	11	2.78–42.56	**0.001**
**Heart SUV at Interim**											
	<median		43	9	21%	1 (ref)			1 (ref)		
	>median		43	1	2%	0.1	0.01–0.80	**0.03**	0.08	0.01–0.63	**0.017**
**Skeletal muscle SUV at Interim**											
	<median		43	4	9%	1 (ref)					
	>median		43	6	14%	1.72	0.48–6.1	0.41			

## Data Availability

The datasets analyzed during the current study are available from the corresponding author on reasonable request.
